# Association between Cardiovascular Diseases and Peri-Implantitis: A Systematic Review and Meta-Analysis

**DOI:** 10.31083/j.rcm2407200

**Published:** 2023-07-13

**Authors:** Danna Chu, Ruiling Wang, Zhen Fan

**Affiliations:** ^1^School & Hospital of Stomatology, Tongji University, Shanghai Engineering Research Center of Tooth Restoration and Regeneration, 200072 Shanghai, China; ^2^Department of Oral Implantology, School & Hospital of Stomatology, Tongji University, Shanghai Engineering Research Center of Tooth Restoration and Regeneration, 200072 Shanghai, China

**Keywords:** dental implants, cardiovascular diseases, epidemiology, inflammation, biomarkers, peri-implantitis

## Abstract

**Background:**

A potential relationship between oral inflammation and cardiovascular disease has been proposed; however, the impact of cardiovascular disease on implant restoration remains unclear. This systematic review aims to assess the relationship between peri-implantitis and cardiovascular disease based on review of data obtained through observational studies.

**Materials and Methods:**

An extensive systematic literature search was performed using the PubMed/MEDLINE, Scopus, Web of Science and Cochrane Library databases. Studies published in English language up to June 2022 were conducted in accordance with PRISMA guidelines. These efforts identified 230 unique publications and, after selection, five studies were included in this meta-analysis. The Newcastle-Ottawa Scale table was used for literature quality assessment. A fixed-effect model was selected and RevMan software version 5.3 was used to identify the origin of the outcomes of the meta-analysis. Finally, results were reported through the PRISMA statement.

**Results:**

This meta-analysis found that in implant restoration the incidence of peri-implantitis in patients with cardiovascular disease was higher than those without cardiovascular disease (Risk ratio (RR) = 1.12; 95% CI: 1.02–1.23; z = 2.34; *p* < 0.05).

**Conclusions:**

Based on current evidence, we conclude that the presence of cardiovascular disease increases the incidence of peri-implantitis.

**Registration:**

PROSPERO database (CRD42022353693).

## 1. Introduction 

Over the past four decades, implant restoration has become one of the most popular approaches for replacing missing teeth [1]. With the goal of promoting early and effective osseointegration, dental implants are superior in terms of patient comfort and longevity, with a long-term survival rate of over 90% [2]. After implant placement, osseointegration with surrounding tissue is required and this is the basis for long-term stability of the implant [3]. However, in the course of clinical practice, there are still technical and biological complications that arise [4], and these can destroy osseointegration and threaten the survival of the implant.

Peri-implantitis, similar to periodontitis, occurring in the tissue surrounding the implant, is believed to be a pathological condition principally associated with dental plaque [5]. The main characteristics of peri-implantitis is the inflammation of the peri-implant mucosa and subsequent progressive loss of supporting bone. Studies have shown that the incidence of peri-implantitis can be as high as 22% (range: 1%–47%) [6], and the control of disease requires early diagnosis and early prevention. According to the third working group of the World Dental Federation Consensus Conference in 2018, Renvert *et al*. [7] summarized the diagnosis of peri-implantitis as follows: (i) Bleeding and/or suppuration on light probing; (ii) increased probing depth compared to baseline; and (iii) bone loss due to initial bone remodeling that exceeds changes at the crest bone level. The occurrence of peri-implantitis is related to many factors. Several studies [8, 9] concluded that history of chronic periodontitis, poor plaque control, and smoking are all risk factors for peri-implantitis. In addition, others [10] have shown that the presence of systemic diseases may have an impact on osseointegration, which is detrimental to the health of implants. However, Diz *et al*. [11] have stated opposing opinions on the existence of systemic diseases. Specifically, these investigators argue that for implant surgery, absolute contraindications are rare, and for systemic diseases, good control is more important than the nature of the disease itself. Recent observations [12] have found that diabetes will result in an increased incidence of peri-implantitis, however, there is no conclusive relationship between cardiovascular disease and peri-implantitis.

Cardiovascular disease (CVD), a class of diseases that occur in the heart and blood vessels, include hypertension, coronary heart disease, and atherosclerosis. From a global perspective, CVD is the leading cause of disease burden [13] and the major reason for mortality, although the survival rates affected by CVD have improved significantly over the past few decades [14]. Domestically, between 1990 and 2016, the age-standardized prevalence of CVD in China increased by 14.7%, and the number of deaths from CVD increased from 2.51 to 3.97 million [15].

More than a century ago, oral sepsis and tooth extraction were considered to be causes of infective endocarditis, and connecting local oral infection with systemic diseases [16]. Furthermore, others have reported that there are certain commonalities between CVD and periodontal disease. For example, periodontitis will lead to the damage of circulating progenitor cells, the number and function of which are markers of endothelial damage related to CVD and are used to evaluate the ability of vascular repair following CVD [17]. At the transcriptional level, periodontitis and periodontal inflamed surface area can predict the level of micro RNAs (miRNAs) associated with subclinical cardiovascular disease risk [18]. More importantly, several studies [19, 20] have shown that patients with CVD with previously diagnosed periodontitis have a significantly increased risk of death. As to the relationship between CVD and peri-implantitis, an inflammatory disease that also occurs in periodontal/peri-implant tissue, a study [21] found that there was a correlation between the risk markers of CVD and peri-implantitis and that high levels of triglycerides and uric acid are not only biochemical markers of CVD but also risk parameters for peri-implantitis. Further examination of the correlation between cardiovascular disease and peri-implant inflammation may prompt clinicians to employ relevant measures prior to the implant operation to improve the success rate of implant and alleviate patient pain. Moreover, this correlation may further support an association between local inflammation and systemic diseases and promote research into this process. Therefore, the present systematic review and meta-analysis aims to augment existing knowledge by including observational studies, such as retrospective and case-control studies to examine the relationship between peri-implantitis and cardiovascular disease.

## 2. Review of Current Literature

### 2.1 Objective

The aim of this study is to screen and analyze epidemiological and observational research studies through a systematic review and meta-analysis to explore the relationship between CVD and peri-implantitis.

### 2.2 Measures

Our research followed the guidelines outlined by Preferred Reporting Items for Systematic review and Meta-Analyses (PRISMA, 2020) [22], and proposed questions in accordance with the principles of PICO (P = population, I = Independent variable, C = comparison, O = outcome measures) [23]:

Population: patients treated with implants restoration.

Independent variable: cardiovascular disease.

Comparison: non-cardiovascular disease.

Outcome: diagnosed with peri-implantitis.

#### 2.2.1 Population

The subjects included in our study were patients who opted for implant restorations for reasons of tooth loss, regardless of jaw position (anterior vs posterior, maxillary vs mandibular), or the number of missing teeth (single vs multiple).

#### 2.2.2 Independent Variables

CVD was selected as an independent variable for several criteria. Specifically, patients undergoing implant surgery suffer from a wide variety of CVDs, including hypertension (systolic blood pressure >140 mmHg, diastolic blood pressure >90 mmHg), coronary heart disease, hyperlipidemia, and cardiomyopathy. Therefore, the term CVD can be used to summarize all diseases occurring in heart and blood vessels.

#### 2.2.3 Comparison

Patients who received implant placement but did not have CVD were included in the control group for analysis.

#### 2.2.4 Outcomes

The outcome selected for this study is peri-implantitis. To unify the pathological criteria for inclusion in the study, we defined peri-implantitis as an inflammatory disease that occurs in the peri-implant tissue with typical inflammatory manifestations and bone loss ≥2 mm on radiographic imaging.

## 3. Materials and Methods

### 3.1 Protocols

#### 3.1.1 Study Registration and Reporting Format

This review is registered in the PROSPERO International Prospective Register of Systematic Reviews and was assigned identification number CRD42022353693. In addition, this study used the twenty-seven preferred reporting items from the 2020 update of the Preferred reporting items for systematic review and meta-analysis (PRISMA) statement to summarize and report the results.

#### 3.1.2 Quality Assessment

Assessment of the quality of the literature included in this study is crucial to the results of the meta-analysis. We evaluated observational experiments in accordance with the Newcastle-Ottawa System (NOS) protocol. The studies included in this paper were all case-control or retrospective cohort studies. Per scoring standards of the NOS scale, each article was awarded a score of 0–9, and those with a score of 5 and above were considered to be high-quality studies.

### 3.2 Search Strategy

We searched PubMed, the Cochrane Library, Scopus and the Web of Science for studies published up to June 2022. The principle of retrieval is to combine the medical subject headings (MESH) terms and their synonyms of keywords within the scope of all fields, so as to make the retrieval accurate and wide-ranging.

**PubMed: 28 hits**

(((Cardiovascular Diseases) OR (Disease, Cardiovascular)) OR (Diseases, Cardiovascular)) AND (((((Peri-Implantitis) OR (Peri Implantitis)) OR (Peri-Implantitides)) OR (Periimplantitis)) OR (Peri implantitides))

**The Cochrane library: 2 hits**

1#:(Cardiovascular Diseases) OR (Disease, Cardiovascular) OR (Diseases, Cardiovascular)

2#:(Peri-Implantitis) OR (Peri Implantitis) OR (Peri-Implantitides) OR (Periimplantitis) OR (Periimplantitides)

3#:1# And 2#

**Scopus: 171 hits**

((cardiovascular AND diseases) OR (disease, AND cardiovascular) OR ( diseases, AND cardiovascular)) AND ((peri-implantitis) OR (peri AND implantitis) OR (peri-implantitides) OR (periimplantitis) OR (periimplantitides )) AND (LIMIT-TO ( DOCTYPE , “ar”)) AND (LIMIT-TO (SUBJAREA , “DENT”))

**Web of science: 71 hits**

(TS = ((Cardiovascular Diseases) OR (Disease, Cardiovascular) OR (Diseases, Cardiovascular))) AND TS = ((Peri-Implantitis) OR (Peri Implantitis) OR (Peri-Implantitides) OR (Periimplantitis) OR (Periimplantitides))

### 3.3 Eligibility Criteria

To ensure the reliability of the included studies, two study investigators screened the literature by title, abstract, or full text. Included studies were required to meet the following requirements:

(1) Explore the relationship between CVD and peri-implantitis.

(2) Reports outlined an observational study such as case-control, cohort studies, and retrospective studies.

(3) Studies indicated a clear diagnosis of peri-implantitis, including clinical and radiological findings.

(4) The follow-up period was not less than 6 months.

(5) The research outlined included not less than 8 patients.

(6) Articles were published in English.

The exclusion criteria are:

(1) Clinical studies with a follow-up time of less than 6 months after implant placement.

(2) Animal experiments, laboratory studies, reviews, meta-analysis, case reports, and case-control studies with less than 8 subjects.

(3) Research published in languages other than English.

### 3.4 Statistical Analysis

Data analysis of the relationship between CVD and peri-implantitis was performed using RevMan software version 5.3 (The Nordic Cochrane Center, The Cochrane collaboration, Copenhagen, Denmark). Risk ratio (RR) values were used to explore the influence of the presence/absence of CVD on the occurrence of peri-implantitis. Heterogeneity was quantified by computing the $I^{2}$ statistical test.

## 4. Results

### 4.1 Study Selection

**Fig. 1.**
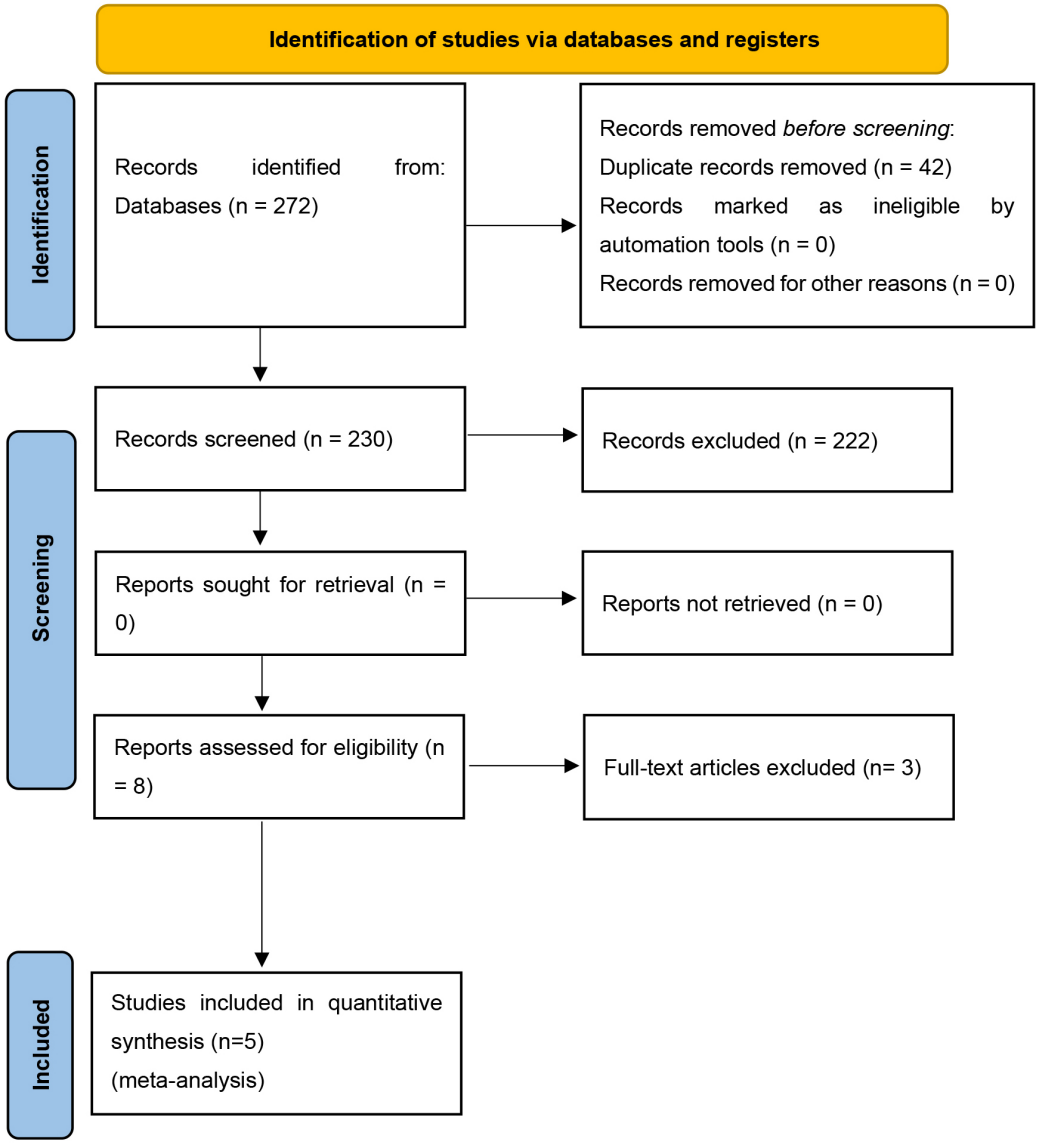
**Identification and selection of eligible studies (PRISMA)**.

We searched four databases and initially obtained 272 records that fit outlined search criteria. After removing duplicates, 230 records were identified (Fig. 1). Based on the titles and abstracts of these 230 articles, 222 records that did not meet our established inclusion criteria were excluded, and the remaining 8 articles underwent full-text review to judge suitability for study inclusion. Three of these eight articles were judged to not meet the inclusion criteria, resulting in five articles selected for inclusion in this meta-analysis [24, 25, 26, 27, 28]. Four of these five studies were case-control studies, also known as retrospective studies, and the other was a retrospective cohort study. A total of 9971 subjects were included in these studies, of which 3214 patients were diagnosed with CVD and the remaining 6757 subjects who received implant restoration had no diagnosis of CVD. These five studies provided prevalence data that could be synthesized by meta-analysis to investigate the relationship between CVD and peri-implantitis.

### 4.2 Study Characteristics

Table 1 (Ref. [7, 24, 25, 26, 27, 28]) summarizes information obtained from the five included studies. The duration of patient follow-up ranged from 26 months to 87.6 months. A total of 9971 patients were included in this meta-analysis, including 5825 (58.7%) female patients and 4146 (41.3%) male patients. The number of patients with pre-existing cardiovascular disease was 3214 (32.23%). Four of these studies provided odds ratio (OR) values for peri-implantitis after implant restoration in patients with CVD, and one study determined the number of implants placed in patients. In addition, the criteria for clinical parameters, such as PD (Probing depth), BOP (Bleeding on probing), RBL (Radiological bone loss), used for the diagnosis of peri-implantitis in each study are listed in Table 1.

**Table 1.** **Main characteristics of included studies**.

	Study design	Durati-on, Mo	Enrolled patients (female/male)	Patients of cardio-vascular diseases	Cardiovascular diseases odds ratio (OR)	Placed implants/Failed implants	Definition of peri-implantitis		
							PD	BOP and/or suppuration	RBL
Revent Stefan *et al*. (2014) [24]	Case-control study	48	270 (161/109)	50	8.7 (*p * < 0.006)	NR	≥4 mm	+	≥2 mm
M. de Araújo Nobre *et al*. (2017) [25]	Retrospective cohort Study	36	8720 (5136/3584)	2845	0.99 (*p* = 0.867)	NR	≥5 mm	+	≥2 mm
J. Neves *et al*. (2018) [26]	Case-control study	87.6	721 (422/299)	222	1.14 (*p* = 0.61)	NR	>4 mm	+	≥2 mm
V. Astolfi *et al*. (2022) [27]	Case-control study	80	132 (55/77)	15	NR	555/0	As Renvert [7] summarized		
I. C. Wang *et al*. (2022) [28]	Case-control study	26	128 (51/77)	82	2.18 (*p* = 0.04)	NR	Increasing	+	≥3 mm

NR, Not Reported; “+”, positive; PD, Probing depth; BOP, Bleeding on probing; RBL, Radiological bone loss.

### 4.3 Association between CVD and Peri-Implantitis

**Fig. 2.**
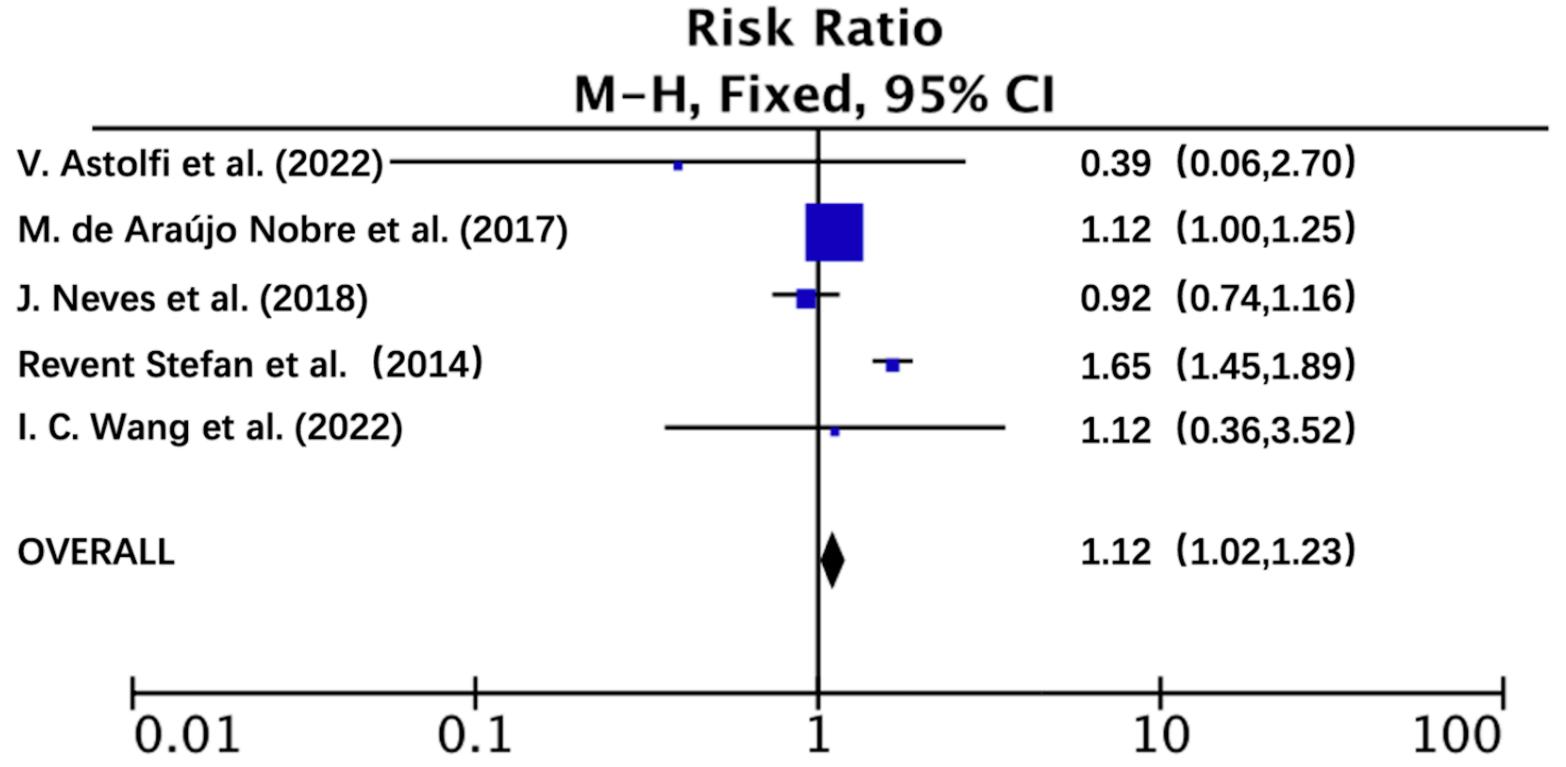
**Forest Plot of the risk of peri-implantitis in cardiovascular diseases compared to non-cardiovascular diseases patients**. RR, risk ratio; CI, Confidence interval; M-H, Mantel-Haenszel.

Analysis of the pooled data using RevMan 5.3 to detect heterogeneity with the dataset indicated that the incidence of peri-implantitis with and without CVD, was different and statistically significant (Chi-square test = 36.40, *p* < 0.05). To further support this conclusion, the data from four case-control studies and one retrospective cohort study were combined and relative risk (RR) values calculated. The results from these analyses are shown in Fig. 2 (RR = 1.12; 95% CI: 1.02–1.23; z = 2.34; *p* < 0.05). The lower and upper bounds and the 95% confidence interval for RR were statistically interpreted and the risk of peri-implantitis in patients with CVD was found to be 1.02 to 1.23 times higher than that in patients without CVD, and that these values reached statistical significance. Fig. 2 shows the RR calculated for each of the five studies, and the pooled RR for the occurrence of peri-implantitis in patients with CVD was found to be 1.12. These findings indicate patients with CVD had a 12% higher risk of developing peri-implantitis after implant restoration compared with patients without CVD.

### 4.4 Quality Assessment and Publication Bias

In sum, we found all articles included in this meta-analysis showed a low risk of bias. Specifically, we calculated a score of more than 5 points using the NOS scale (Table 2, Ref. [24, 25, 26, 27, 28]), thus, all of these reports were included in the meta-analysis. However, most studies did not report the non-response rate of the sample, specifically, the number of patients lost during follow-up. Evidence for publication bias was determined using funnel asymmetry (**Supplementary Fig. 1**).

**Table 2.** **Quality assessment of 5 studies included in the qualitative evaluation according to the modified New Castle Ottawa Scale (NOS)**.

Author (year)	Study design	Section							
		Selection				Comparability	Outcome		
		Case definition	Representativeness of the cases	Selection of Controls	Definition of controls	Control factor	Ascertainment of exposure	Same method of ascertainment for cases and controls	Non-Response rate
Revent *et al*. (2014) [24]	CS	★	NR	★	★	★	★	★	NR
Neves *et al*. (2018) [26]	CS	★	★	★	★	★	★	★	NR
Astolfi *et al*. (2022) [27]	CS	★	★	★	★	★	★	★	NR
Wang *et al*. (2022) [28]	CS	★	★	★	★	★	★	★	NR
Author (year)	Study design	Exposed cohort	Non-exposed cohort	Ascertainment of exposure	Outcome of interest not present at start	Control factor	Assessment of outcome	Follow-up long enough	Adequacy of outcome
de Araújo Nobre *et al*. (2017) [25]	RC	★	★	★	★	★	★	★	NR

NR, Not Reported; “★”, one point; CS, Case-control study; RC, Retrospective cohort study.

## 5. Discussion

This systematic review was performed to summarize the relationship between peri-implant pathology and CVD. Rigorous evaluation of this correlation required a strict definition of peri-implantitis. As outlined above, peri-implantitis was defined as an inflammatory reaction with concomitant loss of supporting bone in tissues surrounding implant. To further improve data reliability, only studies that evaluated the risk of peri-implantitis with and without CVD were included in this meta-analysis. Starting with an initial 272 studies selected using automated approach, the by enforcing outlined inclusion criteria and in-depth analysis of these reports led to the selection of 5 articles for inclusion in this review. Moreover, these included studies had no less than a score of 5 using the NOS scale. The results show that peri-implant inflammation is more likely to occur in patients with CVD.

To our knowledge, this study is the first systematic review and meta-analysis to examine the relationship between peri-implantitis and CVD from the point of view of clinical incidence. However, some comparable information regarding this area is available from other reports. For example, studies have shown a common relationship between CVD and periodontitis [29]. Periodontal disease may have a negative impact on CVD [30], which appears to be linked to the level of circulating progenitor cells. Specifically, other studies determined that circulating endothelial progenitor cells play a significant role in the etiology of periodontitis, and the level of circulating endothelial progenitor cells is inversely correlated with the severity of periodontitis and periodontal disease [17]. In some respects, peri-implantitis is similar to periodontitis, and may share key pathogens such as *Porphyromonas gingivalis, Fusobacterium nucleatum, *and* Tannerella forsythia * [31, 32, 33]. Others [2] showed that in patients with hypertension who underwent implant restoration that although there was no impact on implant survival during the study, there was some adverse effect on clinical parameters of the peri-implant tissue. Such effects included probing depth and the differences were found to be statistically significant. In addition, Wang *et al*. [34] reported the differences between patients with and without CVD and found that the levels of some systemic pro-inflammatory factors such as IL-1β, TNF-α, and MMP-8 were significantly higher in the patients with CVD than those without CVD. Finally, others [35] showed that MMP-8 is an important predictor of periodontitis and peri-implantitis, thus further supporting the relationship between peri-implantitis and CVD.

In a retrospective study by Saaby *et al*. [36], 26% of patients with peri-implantitis, as defined as bone resorption of ≥2 mm and with bleeding or pus on probing, had CVD. In addition, 27.3% of patients with peri-implantitis in the study by Revent *et al*. [24] had CVD, with an OR value of 8.7. These findings indicate that patients with CVD have a significantly higher risk of developing peri-implantitis after implant restoration compared with non-CVD patients. Moreover, these findings are consistent with the results of our study.

In this review, results obtained show an association between peri-implantitis and CVD (RR = 1.12, *p *= 0.02), and that this supports a correlation between local inflammation and the whole-body burden caused by systemic disease. However, our study has some limitations. First, there is a high degree of heterogeneity between selected studies, which may be due to uncontrolled treatment-related factors, such as the number of missing teeth, the three-dimensional position of implantation, and the type of superstructures. Second, none of the five included studies reported the statistical impact on their findings due to lost patient follow-up. Third, potential publication bias stemming from only analyzing studies published in English is possible. Therefore, to obtain firmer conclusions, clinical trials with larger sample sizes, longer follow-up periods, and well-controlled confounders are required.

## 6. Conclusions

The conclusion of this systematic review and meta-analysis clearly indicates that the presence of CVD increases the risk of peri-implantitis. Clinicians need to control CVD as much as possible before implant surgery to reduce the possibility of postoperative complications such as peri-implantitis. Since relevant studies are still limited, additional clinical studies are needed to further refine these conclusions.

## Abbreviations

CVD, Cardiovascular diseases; CS, case-control study; RC, Retrospective cohort study; NR, Not Reported; PD, Probing depth; BOP, Bleeding on probing; RBL, Radiological bone loss.

## Supplementary Material

Supplementary material associated with this article can be found, in the online version, at https://doi.org/10.31083/j.rcm2407200.
