# Supplementary figures and images for: Association between Cardiovascular Diseases and Peri-Implantitis: A Systematic Review and Meta-Analysis
Source: Rev Cardiovasc Med. 2023 Jul 13;24(7):200. doi: 10.31083/j.rcm2407200 (PMC11266458; doi:10.31083/j.rcm2407200)

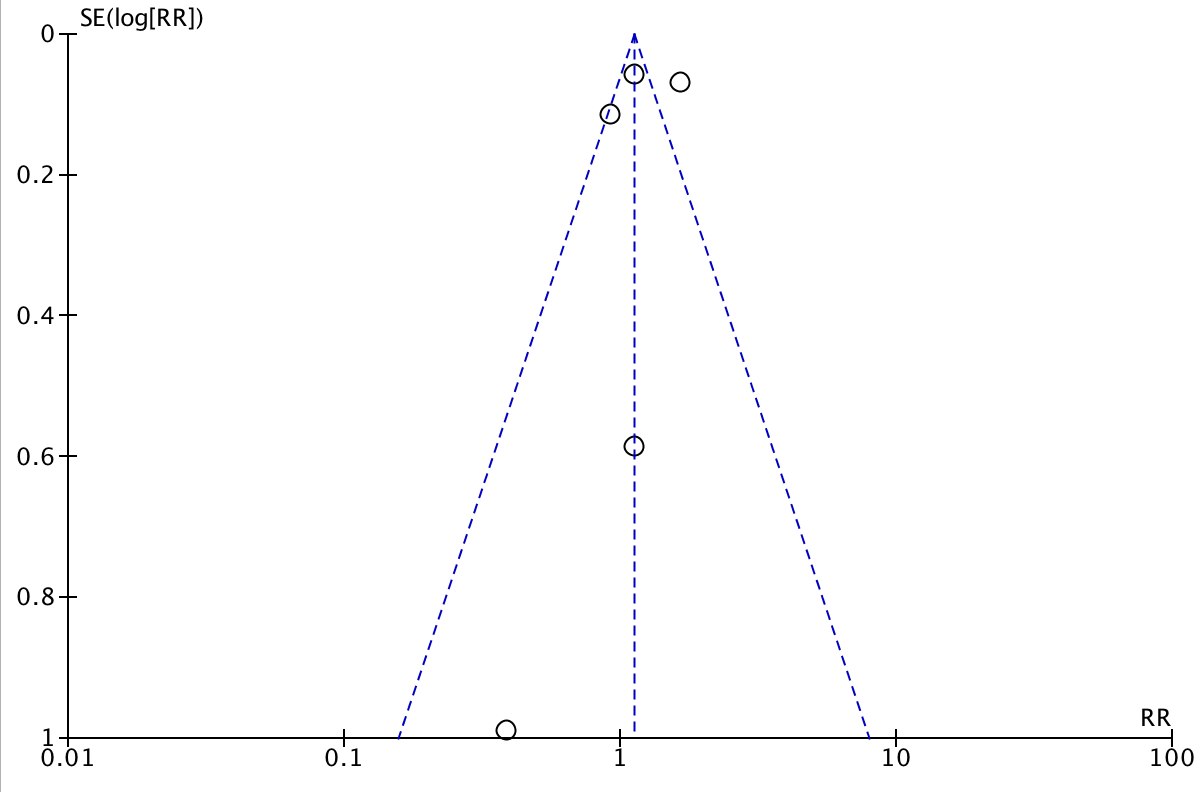


**Fig. 1. Assessment of potential publication bias**

Supplement: Supplementary file 1 [file 2153-8174-24-7-200-s1.zip › Supplementary Material.docx]
